# Screening the Survival of Cyanobacteria Under Perchlorate Stress. Potential Implications for Mars *In Situ* Resource Utilization

**DOI:** 10.1089/ast.2021.0100

**Published:** 2022-06-08

**Authors:** Piotr Rzymski, Barbara Poniedziałek, Natalia Hippmann, Łukasz Kaczmarek

**Affiliations:** ^1^ Department of Environmental Medicine, Poznan University of Medical Sciences, Poznań, Poland.; ^2^ Integrated Science Association (ISA), Universal Scientific Education and Research Network (USERN), Poznań, Poland.; ^3^ Department of Animal Taxonomy and Ecology, Faculty of Biology, Adam Mickiewicz University in Poznań, Poznań, Poland.

**Keywords:** Blue-green algae, Magnesium perchlorate, Mars exploration, Life-support system, Extremophiles

## Abstract

Cyanobacteria are good candidates for various martian applications as a potential source of food, fertilizer, oxygen, and biofuels. However, the increased levels of highly toxic perchlorates may be a significant obstacle to their growth on Mars. Therefore, in the present study, 17 cyanobacteria strains that belong to Chroococcales, Chroococcidiopsidales, Nostocales, Oscillatoriales, Pleurocapsales, and Synechococcales were exposed to 0.25–1.0% magnesium perchlorate concentrations (1.5–6.0 m*M* ClO_4_^−^ ions) for 14 days. The exposure to perchlorate induced at least partial inhibition of growth in all tested strains, although five of them were able to grow at the highest perchlorate concentration: *Chroococcidiopsis thermalis*, *Leptolyngbya foveolarum*, *Arthronema africanum*, *Geitlerinema* cf. *acuminatum*, and *Cephalothrix komarekiana*. *Chroococcidiopsis* sp. *Chroococcidiopsis cubana* demonstrated growth up to 0.5%. Strains that maintained growth displayed significantly increased malondialdehyde content, indicating perchlorate-induced oxidative stress, whereas the chlorophyll *a*/carotenoids ratio tended to be decreased. The results show that selected cyanobacteria from different orders can tolerate perchlorate concentrations typical for the martian regolith, indicating that they may be useful in Mars exploration. Further studies are required to elucidate the biochemical and molecular basis for the perchlorate tolerance in selected cyanobacteria.

## 1. Introduction

Space exploration is driven by curiosity regarding Earth's place in the Universe, scientific interests, a better understanding of past and future Earth impacts, the potential to extract natural resources from extraterrestrial bodies and develop high-tech solutions to current problems, and concern about the long-term future of humanity on Earth (Bainbridge, 2009; Launius, 2019). Although the Kepler space observatory, launched in 2009 and followed by the K2 Mission, offered an unprecedented opportunity to reach beyond the Solar System and identify exoplanets and multi-planet systems, current technology does not allow direct exploration of these exosolar worlds (Christiansen *et al.*, 2020; Saha *et al.*, 2020).

However, exciting opportunities have come to light with regard to the search for extraterrestrial life within the Solar System and the survival of selected terrestrial organisms, known as extremophiles, under simulated conditions of planets such as Mars (Kounaves, 2007; Direito *et al.*, 2011; Mastascusa *et al.*, 2014; Merino *et al.*, 2019).

Although it is possible that some organisms may have inhabited Mars in the past, Mars' current surface conditions, especially the highly oxidative atmosphere, the flux of UVB and UVC, the low temperatures, and the xeric environment, are considered prohibitive with regard to most known life-forms (Read *et al.*, 2015; Martínez *et al.*, 2017). A number of experiments have been conducted to assess whether selected terrestrial organisms have the potential to thrive under current martian physical and chemical conditions (Berry *et al.*, 2010; de Vera, 2012; Frösler *et al.*, 2017). Interestingly, selected cyanobacteria that have adapted to extreme environments on Earth and vary in their response to pH, temperature, salinity, humidity, ultraviolet (UV) radiation, and pollution have exhibited such capabilities (Grilli Caiola and Billi, 2007; Billi, 2008; Olsson-Francis *et al.*, 2010).

These investigations have highlighted cyanobacteria as a potential biological component of life-support systems for manned space missions to Mars (Verseux *et al.*, 2016). As has been argued, resources required to sustain cyanobacteria are available on Mars, and cyanobacteria would offer a number of benefits that include (i) oxygen production via photosynthesis that, for some species, can be efficiently maintained under conditions of low light (Nürnberg *et al.*, 2018), (ii) the formation of organic nitrogen for potential use by other life-forms via the diazotrophic metabolism exhibited by some filamentous species (Stal, 2015), (iii) serving as a source of food because some species are edible, nontoxic, and offer high protein content and high digestibility (Niccolai *et al.*, 2019), (iv) serving as a fertilizer for plant growth in food production units (Jhala *et al.*, 2017), (v) serving as a nutrient source for other microorganisms (*e.g.,* yeasts) or microinvertebrates via lysed biomass (Möllers *et al.*, 2014), (vi) serving as a platform for the production of biofuels such as biohydrogen, biomethane, bioethanol, and biodiesel (Farrokh *et al.*, 2019), and (vii) production of biological crusts via the release of particle-binding polysaccharides that can provide protection from martian surface dust (Liu *et al.*, 2008).

Altogether, the unique features of cyanobacteria make them good candidates for a variety of martian applications (Verseux *et al.*, 2016). In particular, the unicellular polyextremophile *Chroococcidiopsis* sp. has been suggested as one of the most promising cyanobacteria for Mars exploration due to its resistance to multiple stressors that include desiccation, UV and ionizing irradiation, a wide range of temperatures, and low requirements for nutrients and light availability (Grilli Caiola *et al.*, 1993; Friedmann and Ocampo-Friedmann, 1995; Billi and Grilli Caiola, 1996; Billi *et al.*, 2000).

Moreover, a culture of *Chroococcidiopsis* sp. survived exposure to outer space for 548 days at the International Space Station (Cockell *et al.*, 2011). This species could also withstand the low atmospheric pressure of Mars and high martian UV flux if buried 1 mm under the surface of a martian regolith analogue (Cockell *et al.*, 2005; Baqué *et al.*, 2013a).

The relatively recent discovery of perchlorate (ClO_4_^−^) in martian regolith is considered a significant challenge for the survival of terrestrial life-forms on the surface of Mars. NASA's Phoenix lander first detected these highly reactive chemicals in 2008 (Hecht *et al.*, 2009; Kounaves *et al.*, 2010, 2014). Its presence was later confirmed by the Sample Analysis at Mars instrument on the NASA's Curiosity rover in the region of Gale Crater (Glavin *et al.*, 2013; Leshin *et al.*, 2013; Ming *et al.*, 2014; Sutter *et al.*, 2017; Martin *et al.*, 2020). Finally, the presence of hydrated salts of Mg(ClO_4_)_2_, Mg(ClO_3_)_2_, and NaClO_4_ was identified spectroscopically by the Mars Reconnaissance Orbiter in 2015 in locations believed to be characterized by the presence of brine (Ojha *et al.*, 2015).

Altogether, these observations suggest that martian perchlorates are ubiquitously and globally distributed. Their expected levels, which reach a mean of 0.6 wt % (Hecht *et al.*, 2009; Kounaves *et al.*, 2010), are toxic to Earth organisms (Kumarathilaka *et al.*, 2016; Pleus and Corey, 2018) and have been shown to exhibit a significant bactericidal effect and magnify the adverse action of UV irradiation (Anderson *et al.*, 2000; Wadsworth and Cockell, 2017). On the other hand, selected bacteria, such as *Azospirillum* spp., *Dechloromonas* spp., and *Dechlorosoma* spp., utilize perchlorates as a terminal electron acceptor and execute its effective reduction via the enzyme perchlorate reductase (Bender *et al.*, 2005; Nozawa-Inoue *et al.*, 2005; Carlström *et al.*, 2015).

To date, there is no evidence that any cyanobacteria species are capable of this process, and it remains to be investigated whether they can thrive under high perchlorate concentrations. Clarification of this matter is pivotal for the understanding of the scope of application of cyanobacteria in Mars exploration.

In the present study, we screened the potential for 17 cyanobacteria that are associated with distinctively different habitats and represent different orders (Chroococcales, Chroococcidiopsidales, Nostocales, Oscillatoriales, Pleurocapsales, and Synechococcales) to survive in 0.25–1.0% perchlorate, which are concentrations that can be expected in the martian regolith. The cultures' growth, pigment ratio, and lipid peroxidation (a marker of oxidative stress) were monitored over a 2-week period. The present study provides an insight into the cyanobacterial strains that demonstrate a degree of tolerance to perchlorate, a promising feature in light of the potential use of these microorganisms in *in situ* resource utilization for Mars life support systems.

## 2. Materials and Methods

### 2.1. Cyanobacteria strains

A total of 17 strains that originated from aquatic and terrestrial habitats that differ in ambient conditions were used. All were obtained from the Culture Collection of Autotrophic Organisms (CCALA, Třeboň, Czech Republic), except for *Microcystis aeruginosa* (Kützing, 1846) (strain SAG 14.85), which was purchased from the Culture Collection of Algae at Goettingen University (Germany). A detailed list of the cyanobacteria used and their main characteristics is presented in Table 1.

**Table 1.** The General Characteristics of the Cyanobacteria Strains Tested in the Present Study

Species/strain	Order	Origin	Habitat	Morphological form	GenBank (16S rRNA)
*Chalicogloea cavernicola* Roldán *et al.*, 2012 (M.HERNANDEZ MARINE 2011/1)	Chroococcales	Spain	Cave	Unicellular	JQ967037
*Chroococcus* cf. *membraninus* (CCAP 1412/5)		Unknown	Thermal spring	Unicellular	GQ375049
*Microcystis aeruginosa* Kützing, 1846 (SAG 14.85)		Little Rideau Lake, Ontario, Canada	Freshwater	Unicellular, colonial	AJ133171 NZ_CAIK00000000 (whole genome)
*Chroococcidiopsi*s sp. (HINDAK 1968/64)	Chroococcidiopsidales	Mamaia, Constanta, Romania	Psammon	Unicellular, forming agglomerate packets	MH208397
*Chroococcidiopsi*s *cubana* Komárek and Hindák, 1975 (HINDAK 1965/21)		Santa Fe, Cuba	Mineral spring, stone		MH208402
*Chroococcidiopsis thermalis* Geitler, 1933 (HINDAK 1975/67)		Piestany, Slovakia	Thermal mud		MH208404
*Anabaena laxa* (Braun) Bornet and Flahault, 1886 (TAKACOVA 1991/1)	Nostocales	Czech Republic	Soil	Filamentous	—
*Calochaete cimrmanii* Hauer *et al.*, 2013 (MÜHLSTEINOVÁ/2013/1)		Chirripó Mountain, Costa Rica	Aerophitic on rock		HF912386
*Cephalothrix komarekiana* Malone *et al.,* 2015 (SAG 75.79)	Oscillatoriales	Mallorca, Spain	Concrete	Filamentous	EF654083
*Geitlerinema* cf. *acuminatum* (HINDAK 1967/39)		Brasov, Romania	Soil		EU196627
*Kastovskya adunca* (Schwabe) Mühlsteinová *et al.*, 2014 (ATA3-4Q-CV17)		Atacama Desert, Chile	Quartz rocks		KF312342
*Myxosarcina* sp. (HINDAK 1969/25)	Pleurocapsales	Ohrid, Macedonia	Freshwater	Unicellular, colonial	—
*Myxosarcina* sp. (HINDAK 1981/15)		Fishpond, Trnava, Slovakia	Freshwater		—
*Pleurocapsa* sp.		Vysoke Tatry, Slovakia	Freshwater		NZ_PVWF01000000 (whole genome)
*Aphanocapsa rivularis* (Carmichael) Rabenhorst, 1865 (CCAP 1404-1)	Synechococcales	United Kingdom		Unicellular	—
*Arthronema africanum* (Schwabe and Simonsen) Komárek and Lukavský, 1988 (LUKAVSKY 1980/1)		Kuwait	Salt marsh	Filamentous	MT741855
*Leptolyngbya foveolarum* (Gomont) Anagnostidis and Komárek, 1988 (HINDAK 1963/111)		Herculaneum, Italy	Soil	Filamentous	—

### 2.2. Experimental design

The experiments were designed to screen the ability of different cyanobacteria to survive under increased perchlorate concentrations under otherwise optimal conditions. The cyanobacteria were harvested at the late log growth phase and incubated at low density (OD_750_ = 0.035–0.060) in 250 mL culture flasks containing 50 mL of fresh, sterile media (Z-medium or BG-11 depending on the recommendations for a particular strain) with magnesium perchlorate added to reach a final concentration of 0.25, 0.5, or 1.0% containing 1.5, 3.0, and 6.0 m*M* ClO_4_^−^ ions, respectively. The control samples consisted of culture incubated in optimal medium without the addition of perchlorates.

Magnesium perchlorate [Mg(ClO_4_)_2_; Sigma-Aldrich, Germany] was selected for the experiments given that its presence in martian regolith was spectroscopically confirmed by the Mars Reconnaissance Orbiter (Ojha *et al.*, 2015). Cyanobacterial cultures were grown for 14 days at 21°C under 80 μmol/(m^2^·s^2^) irradiance by using cool white fluorescent light with a photoperiod regime of 12 h dark and 12 h light. Each culture was shaken manually twice daily. Three independent replicates of each experiment were carried out. An Institutional Review Board approval was not required for this type of experiment.

### 2.3. Growth analysis

The growth kinetics in perchlorate-exposed and control cultures were monitored with a Synergy HTX Multi-Mode Microplate Reader (BioTek) with OD_750_ (Moheimani *et al.*, 2013) at baseline and 1, 3, 5, 7, and 14 days after incubation.

### 2.4. Lipid peroxidation assay

Cultures that displayed growth at the end of the experiment were subjected to a lipid peroxidation assay with malondialdehyde (MDA) as a surrogate (Rzymski *et al.*, 2020). To this end, a TBARS Assay Kit (Cayman Chemical) was employed. After the incubation, cells were collected from a 5 mL subsample of each culture by centrifugation, washed twice with fresh medium (Z medium or BG-11), and incubated for 30 min at 21°C with gentle shaking on an orbital shaker with a 1% Triton X-100 cell-lysis buffer (Cayman Chemical). Butylated hydroxytoluene was added to prevent artificial lipid peroxidation.

Samples were then centrifuged (1600 *g*, 10 min, 4°C) to remove insoluble material. The protein content in supernatants (5 μL) was quantified with a Quick Start™ Bradford Protein Assay Kit (Bio-Rad) by using the microassay method and bovine serum albumin as a protein standard. To generate MDA adducts with thiobarbituric acid (TBA), 100 μL of supernatant was mixed with 800 μL of TBA. After incubation at 95°C for 60 min, samples were placed in an ice bath for 10 min to stop the reaction and centrifuged (1600 *g*, 10 min, 4°C). The absorbance of the supernatants was measured at 532 nm, compared with a calibration curve prepared by using an MDA standard (Cayman Chemical), and reported as micromole MDA per gram protein.

### 2.5. Pigment analysis

After incubation, the concentrations of chlorophyll *a* and total carotenoid pigments were measured in surviving cyanobacteria that displayed growth after 14 days of exposure to perchlorate. To this end, the spectrophotometric method developed by Zavřel *et al.* (2015) was employed. Briefly, 1 mL of each culture was centrifuged at 15,000 g for 1 mL, and the pellet was extracted with cold methanol and centrifuged again. The absorbance (A) was measured at 470, 665, and 720 nm. The chlorophyll *a* concentration was calculated by using the formula:

$$\begin{array}{c} Chlorophylla\left[\mu g∕mL\right]=12.9447\left(A665-A720\right)\\ \left(Ritchie,2006\right). \end{array}$$

Carotenoid content was calculated by using the formula:


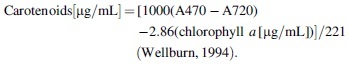


The chlorophyll *a*/carotenoids ratio was derived from the calculated concentrations.

### 2.6. Statistical analysis

The statistical analyses were conducted with STATISTICA 13.0 (StatSoft, Tulsa, OK). Because not all the data met the assumption of Gaussian distribution, nonparametric methods were employed. To test differences between control and experimental groups, the Mann–Whitney *U* test (comparison of two groups) or the Kruskal–Wallis ANOVA with the *post hoc* Dunn's test (comparison of more than two groups) was employed. A value of *p* < 0.05 was considered as statistically significant.

## 3. Results

### 3.1. Growth

**FIG. 1.**
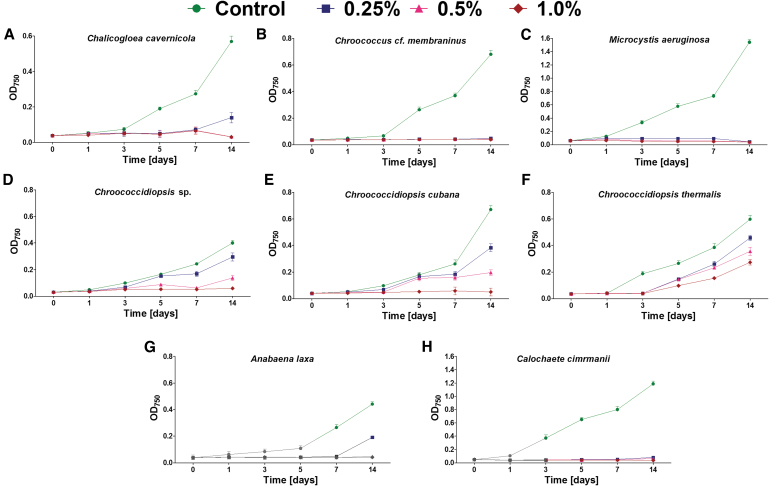
The growth (mean and SD) of cyanobacteria belonging to Chroococcales **(A–C)**, Chroococcidiopsidales **(D–F)**, and Nostocales **(G, H)** orders exposed to different concentrations of magnesium perchlorate (*n* = 3). SD = standard deviation. Color graphics are available online.

**FIG. 2.**
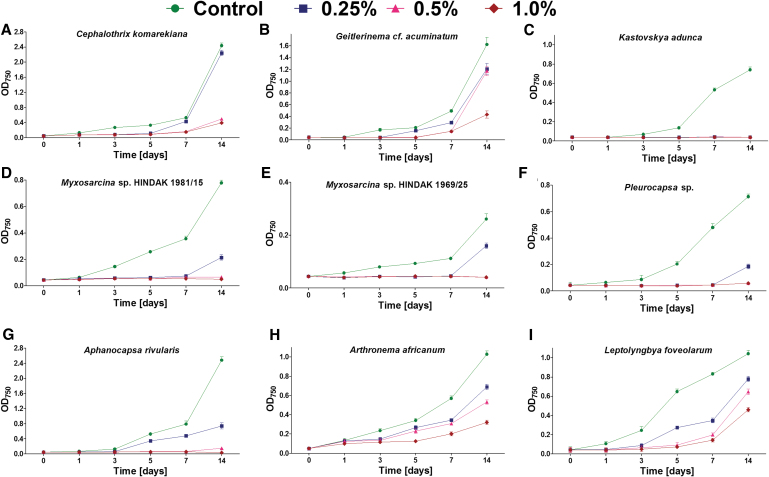
The growth (mean and SD) of cyanobacteria belonging to Oscillatoriales **(A–C)**, Pleurocapsales **(D–F)**, and Synechococcales **(G–I)** orders exposed to different concentrations of magnesium perchlorate (*n* = 3). Color graphics are available online.

Varying kinetics of cyanobacteria growth were observed under the perchlorate exposure (Figs. 1 and 2). Generally, three groups could be distinguished as follows:

(i) No growth in the presence of any tested magnesium perchlorate concentration: *Chroococcus* cf. *membraninus*, *M. aeruginosa*, *Calochaete cimrmanii*, *Kastovskya adunca*.
(i i) Growth only at lower magnesium perchlorate concentrations: *Chroococcidiopsis* sp. (at 0.25–0.5%), *Chroococcidiopsis cubana* (at 0.25–0.5%), *Chalicogloea cavernicola* (at 0.25%), *Anabaena laxa* (at 0.25%), both *Myxosarcina* sp. strains (at 0.25%), *Pleurocapsa* sp. (at 0.25%), and *Aphanocapsa rivularis* (at 0.25%).
(i ii) Growth in all magnesium perchlorate concentrations (0.25–1.0%), but at different growth rates: *Chroococcidiopsis thermalis*, *Cephalothrix komarekiana*, *Geitlerinema* cf. *acuminatum*, *Arthronema africanum*, and *Leptolyngbya foveolarum*.

Despite some strains maintaining growth during the 14 days of exposure, at least partial inhibition was observed in all cases and under all perchlorate concentrations (Figs. 1 and 2). The most tolerant strains included *C. thermalis* (Fig. 1F) and *L. foveolarum* (Fig. 2I), for which the exposure to 0.25% of magnesium perchlorate resulted in growth at a level of 77% and 75% of control, respectively. In comparison, at 0.5% and 1.0% concentration, the *C. thermalis* grew at a rate of 60% and 46% of control, respectively, whereas *L. foveolarum* grew at a rate of 62% and 44%.

In the case of *A. africanum*, exposure to 0.25%, 0.5%, and 1.0% magnesium perchlorate concentration caused growth at 67%, 52%, and 31% of control, respectively (Fig. 2H); for *G.* cf. *acuminatum*, the same exposure caused growth at 74%, 72%, and 27% (Fig. 2H), respectively; and for *C. komarekiana*, the same exposure caused growth at 92%, 20%, and 16%, respectively (Fig. 2A).

### 3.2. Lipid peroxidation

**FIG. 3.**
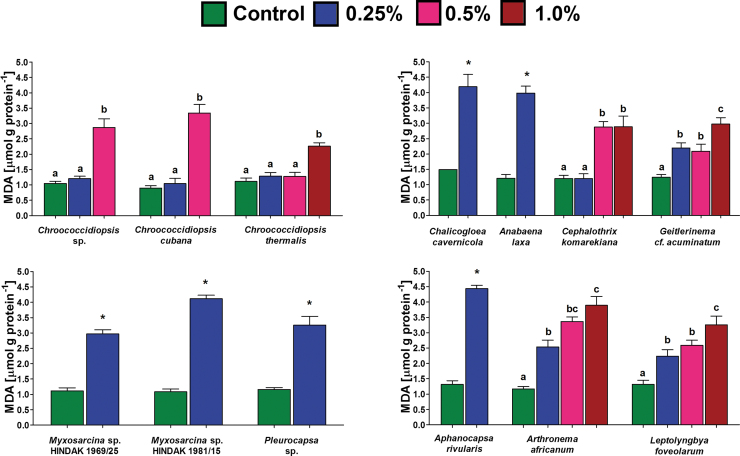
The lipid peroxidation measured by means of intracellular thiobarbituric acid reactive substance content (mean and SD), represented predominantly by MDA in selected cyanobacteria that displayed growth after 14 days of exposure to different magnesium perchlorate concentrations. An asterisk indicates a significant difference with the control (*p* < 0.05; Mann–Whitney *U* test). Different letters in the column indicate statistically significant differences between the samples obtained for each species demonstrated with the *post hoc* Dunn's test following the Kruskal–Wallis ANOVA (*p* < 0.05). MDA, malondialdehyde. Color graphics are available online.

The exposure of cyanobacteria to perchlorates was shown to induce lipid peroxidation (Fig. 3). Compared with the control, most of the cultures that maintained growth for the entire 14 days revealed a significantly increased content of MDA. However, in three strains that belonged to the genus *Chroococcidiopsis*, no differences were seen up to a 0.5% concentration. For *C. komarekiana*, a significant increase was noted at levels of 0.5% and 1.0%. In general, species that maintained growth only at 0.25% concentration of magnesium perchlorate (*A. laxa*, *A. rivularis*, *C. cavernicola*, both *Myxosarcina* sp. strains, *Pleurocapsa* sp.) revealed the highest intracellular levels of MDA (Fig. 3).

### 3.3. Pigment content and ratio

In general, the perchlorate-exposed cyanobacterial cultures, for which the growth was maintained after 14 days, displayed a significant decrease in chlorophyll *a* and total carotenoid content in a concentration-dependent manner. However, in the case of *C. thermalis* and *L. foveolarum*, a respective 40–60% and 25% increase in carotenoid content was noted under perchlorate exposure. For the majority of cultures, the chlorophyll *a*/carotenoid ratio decreased, except for *Chroococcidiopsi*s sp. and *Myxosarcina* sp. (HINDAK 1969/25), for which an increase was observed (Table 2).

**Table 2.** The Concentration (Mean ± Standard Deviation) of Chlorophyll *a* and Total Carotenoids (μg/mL) and Their Ratio in Magnesium Perchlorate-Exposed Cyanobacteria Displaying Growth After 14 Days

Species/strain	Sample	Chlorophyll a	Total carotenoids	Ratio chlorophyll* a*/carotenoids
*Chalicogloea cavernicola*	Control	5.5 ± 0.1	1.2 ± 0.1	4.6
	0.25%	3.4 ± 0.1	1.1 ± 0.1	3.1^*^
*Chroococcidiopsi*s sp.	Control	4.2 ± 0.1	1.8 ± 0.1	2.3^a^
	0.25%	2.4 ± 0.5	1.2 ± 0.2	2.0^a^
	0.5%	1.8 ± 0.1	0.5 ± 0.1	3.6^b^
*Chroococcidiopsis cubana*	Control	4.5 ± 0.1	1.6 ± 0.1	2.8^a^
	0.25%	3.8 ± 0.1	1.0 ± 0.1	3.8^b^
	0.5%	1.3 ± 0.3	0.6 ± 0.1	2.2^a^
*Chroococcidiopsis thermalis*	Control	4.8 ± 0.1	0.5 ± 0.1	9.6^a^
	0.25%	3.8 ± 0.1	0.8 ± 0.1	4.7^b^
	0.5%	2.5 ± 0.1	0.8 ± 0.1	3.1^b^
	1.0%	1.4 ± 0.2	0.7 ± 0.2	2.0^c^
*Anabaena laxa*	Control	4.3 ± 0.1	1.4 ± 0.1	3.1
	0.25%	3.1 ± 0.2	1.0 ± 0.1	3.1
*Cephalothrix komarekiana*	Control	17.6 ± 0.4	4.1 ± 2.7	4.3^a^
	0.25%	11.3 ± 0.1	4.8 ± 0.2	2.4^b^
	0.5%	6.7 ± 0.2	3.1 ± 0.1	2.2^b^
	1.0%	4.5 ± 0.2	2.0 ± 0.1	2.2^b^
*Geitlerinema* cf. *acuminatum*	Control	5.2 ± 0.1	1.4 ± 0.1	3.7^a^
	0.25%	4.1 ± 0.2	1.1 ± 0.1	3.7^a^
	0.5%	4.2 ± 0.2	0.8 ± 0.1	5.2^b^
	1.0%	3.0 ± 0.1	0.9 ± 0.2	3.3^a^
*Myxosarcina* sp. HINDAK 1969/25	Control	4.0 ± 0.1	2.0 ± 0.1	2.0
	0.25%	2.5 ± 0.1	1.5 ± 0.1	1.7
*Myxosarcina* sp. HINDAK 1981/15	Control	3.2 ± 0.1	1.2 ± 0.1	2.7^b^
	0.25%	2.3 ± 0.1	0.7 ± 0.1	3.3^b^
*Pleurocapsa* sp.	Control	4.5 ± 0.1	1.3 ± 0.1	3.5
	0.25%	3.4 ± 0.1	1.1 ± 0.1	3.1^*^
*Aphanocapsa rivularis*	Control	7.5 ± 0.1	1.8 ± 0.1	4.2
	0.25%	4.1 ± 0.2	1.5 ± 0.1	2.7^*^
*Arthronema africanum*	Control	8.7 ± 0.2	2.9 ± 0.1	3.8^a^
	0.25%	7.0 ± 0.1	2.3 ± 0.1	3.0^b^
	0.5%	5.2 ± 0.1	1.9 ± 0.1	2.7^b^
	1.0%	2.1 ± 0.2	0.8 ± 0.1	2.6^b^
*Leptolyngbya foveolarum*	Control	4.2 ± 0.1	1.6 ± 0.1	2.6^a^
	0.25%	4.1 ± 0.1	1.5 ± 0.1	2.7^a^
	0.5%	3.1 ± 0.1	1.5 ± 0.1	2.1^b^
	1.0%	2.5 ± 0.1	2.0 ± 0.2	1.3^c^

Different letters in the column indicate statistically significant differences between the samples obtained for each species demonstrated with the *post hoc* Dunn's test following the Kruskal–Wallis ANOVA (*p* < 0.05).

^*^ Indicates a difference with control (Mann–Whitney *U* test, *p* < 0.05).

In the case of strains that survived the highest, 1.0% concentration of magnesium perchlorate, the most significant decrease in the pigment ratio was observed for *C. thermalis* (4.8-fold) followed by *L*. *foveolarum* (2.0-fold), *C*. *komarekiana* (1.9-fold), *A*. *africanum* (1.5-fold), and *G*. cf. *acuminatum* (1.1-fold).

## 4. Discussion

The present study is a pilot screening for the survival of different cyanobacteria when exposed to increased perchlorate concentrations. Out of the 17 strains tested, 13 (76.5%) demonstrated at least a partial tolerance to perchlorates, whereas 5 were capable of maintaining growth at the highest tested 1% concentration of magnesium perchlorate that contained 6.0 m*M* of ClO_4_^−^ ions. These findings are important in light of the potential use of cyanobacteria in Mars exploration since the martian surface is characterized by increased levels of perchlorate salts, which are, in turn, extremely soluble. Surviving under such chemicals indicates that cyanobacteria could be useful as pioneer microorganisms in terraforming Mars, biomining of basalt and potential ores on Mars, or processing regolith into a substrate utilized by other organisms.

As expected, cyanobacteria strains that belong to the order Chroococcidiopsidales demonstrated tolerance to perchlorates as previously shown for *Chroococcidiopsis* sp. (CCMEE 029) (Billi *et al.*, 2021). In the present study, *C. thermalis* was the most tolerant of all tested strains of cyanobacteria, maintaining growth throughout the 0.25–1.0% magnesium perchlorate concentration range. Two other tested members of the genus *Chroococcidiopsis*, that is, *Chroococcidiopsi*s sp. (HINDAK 1968/64) and *C. cubana*, thrived in a magnesium perchlorate concentration of up to 0.5%.

Previous studies have shown that different *Chroococcidiopsis* strains can differ in tolerance to various conditions such as desiccation, which is a feature related to their antioxidant system efficiency (Fagliarone *et al.*, 2017). Altogether, the present study confirms that the Chroococcidiopsidales are probably the most promising group of cyanobacteria for martian use, especially given that their resistance to various other extreme conditions (*e.g.,* ionizing and UVC radiation, desiccation) has been documented in previous studies (Grilli Caiola *et al.*, 1993; Baqué *et al.*, 2013b; Verseux *et al.*, 2017).

Other cyanobacteria that survived and displayed growth after 14 days of exposure to the highest perchlorate concentration included terrestrial species associated with soil (*G.* cf. *acuminatum, L. foveolarum*), concrete (*C. komarekiana*), and salt marshes (*A. africanum*). It is plausible that all of them share some degree of resistance to desiccation and UV radiation—the latter was just recently confirmed for *C. komarekiana* (Hossain *et al.*, 2021)—both of which are factors that can induce significant oxidative stress (Santos *et al.*, 2012). Studies of desiccation-tolerant bacteria such as *Hydrogenothermus marinus* (Stohr *et al.*, 2001) have demonstrated that it can grow under a high concentration of perchlorate (Beblo-Vranesevic *et al.*, 2017).

On Earth, the direct adaptation to perchlorates in cyanobacteria could only evolve in a few environments, since naturally occurring perchlorates are generally restricted to evaporites in hyperarid regions such as the Atacama Desert (Ericksen, 1983). However, it should be noted that their reported content in such areas ranges from 290 to 2500 μg/kg (0.000029–0.00025%) (Calderón *et al.*, 2014), which is a 1000 times less than the perchlorate levels used in the present study.

Surprisingly, *K. adunca*, a strain isolated from the Atacama Desert, did not reveal any resistance to perchlorates in the present study. Whether the studied cyanobacteria can also reduce perchlorates, as observed for bacteria associated with hypersaline soils (Acevedo-Barrios *et al.*, 2019), remains to be demonstrated (Table 3). To the best of our knowledge, the expression of perchlorate reductase and chlorite dismutase, enzymes that carry out the reduction or elimination of perchlorates (Xu and Logan, 2003; Bender *et al.*, 2005), has never been studied in cyanobacteria.

**Table 3.** Hypotheses on the Perchlorate Tolerance in Selected Cyanobacteria and Research Needed for Their Testing

Hypothesis	Research needed
Selected cyanobacteria are metabolizing perchlorates and subsequently decreasing their toxicity	Molecular studies on the expression of perchlorate reducing enzymes in cyanobacteria Enzymatic activity studies of cyanobacteria Analytical studies of the kinetics of perchlorate concentration in cyanobacteria culture
Selected cyanobacteria are surviving perchlorate exposure due to overexpression of factors protecting from perchlorate-induced oxidative stress and related cellular damage	Real-time monitoring of intracellular reactive oxygen species levels under perchlorate stress Molecular studies of the expression of genes involved in the oxidative stress response
Selected cyanobacteria are releasing compounds that interact with perchlorates and reduce their toxicity	Analytical studies of the kinetics of perchlorate concentrations in solutions containing cyanobacterial exudates/lysates or spent cyanobacterial culture medium. Studies on the effect of known cyanobacterial metabolites on perchlorate concentrations
Perchlorates are inducing noncritical damage in selected cyanobacteria, but limiting the growth of cultures	Examination of the morphology of cyanobacteria cells under perchlorate stress Examination of cell membrane integrity using fluorescence probe (*e.g.,* SYTOX Green) and fluorescent microscopy/flow cytometry

The present study also confirmed that perchlorate exposure leads to oxidative stress, given that an increased level of lipid peroxidation was observed for all cyanobacteria that maintained growth after 2 weeks of exposure. In general, the species that survived the highest tested concentration of perchlorate had lower levels of peroxidation of lipids compared with those that only survived at lower concentrations. Altogether, this indicates that the tolerance to perchlorates in cyanobacteria is at least partially related to antioxidant capacities.

This ability may be related to the enzymatic antioxidant system based on superoxide dismutase (SOD) and catalase and the cellular content of nonenzymatic compounds such as reduced glutathione and phenolics (Latifi *et al.*, 2009). As previously shown, cyanobacteria that belong to the genera *Leptolyngbya* and *Chroococcidiopsis* are rich in flavonoids (Ijaz and Hasnain, 2016). Further, *Chroococcidiopsis* species have demonstrated the overexpression of genes encoding SODs under oxidative stress induced by desiccation (Napoli *et al.*, 2021).

It was also experimentally shown that various strains of *Chroococcidiopsis* reveal different resistance to desiccation and radiation that correlates with avoidance of protein carbonylation, a marker for oxidative damage to proteins promoted by multiple reactive oxygen species (Fagliarone *et al.*, 2017). The present study confirmed that *Chroococcidiopsis* species are highly resistant to perchlorate-induced oxidative stress and display no difference in intracellular MDA content after 14 days of exposure to concentrations of up to 0.5%.

In general, this confirms that perchlorate exposure can lead to oxidation damage in cyanobacteria, the avoidance of which is related with some degree of perchlorate tolerance. It is, therefore, likely that *C*. cf. *membraninus*, *C*. *cimrmanii*, *K*. *adunca*, and *M*. *aeruginosa* do not cope well enough with oxidative stress to survive perchlorate exposure. In line with this, previous studies have shown that *M*. *aeruginosa* is susceptible to various toxic chemicals due to oxidative damage that ultimately results in significant growth inhibition (Li *et al.*, 2016; Rzymski *et al.*, 2020; Zheng *et al.*, 2021).

Considering that perchlorate exposure was associated with increased oxidative stress, the observed shift in carotenoid content in relation to the level of chlorophyll *a* can also represent the protective response. Importantly, the two most tolerant species, *C. thermalis* and *L. foveolarum*, revealed an increase in carotenoid content under perchlorate exposure compared with the control. Carotenoids have the ability, among various roles, to inhibit free radical reactions, which mitigate oxidative stress (Gao and Garcia-Pichel, 2011; Zakar *et al.*, 2016).

Their accumulation in *Chroococcidiopsis* sp. has been previously associated with protection against the toxic effects of UVC exposure (Baqué *et al.*, 2013b). Moreover, it was also shown that in *Chroococcidiopsis* sp., carotenoids are resistant to oxidation and subsequent degradation when exposed to high doses of gamma irradiation (Baqué *et al.*, 2020). It would be of interest to further study the exact composition of carotenoids in perchlorate-resistance cyanobacteria. It has been suggested that carotenoids, with a high degree of unsaturated bonds and the glycosidic nature, for example, myxoxanthophyll, are particularly effective in protection from oxidative damage such as peroxidation (Steiger *et al.*, 1999; Latifi *et al.*, 2009).

Perchlorates are known not to be readily susceptible to chemical degradation, although some compounds, for example, ferric chloride or hydrochloric acid, are known to degrade them nearly completely (Gu *et al.*, 2003; Brusseau, 2019). Cyanobacteria are known to produce a vast array of secondary metabolites (alkaloids, nonribosomal and ribosomal peptides, polyketides, isoprenoids; for a comprehensive list, see an open CyanoMetDB database), some of which are actively released by intact cells, whereas others are released during cell death (Jones *et al.*, 2021).

Whether any of these cyanometabolites could facilitate perchlorate degradation (*e.g.,* reduction to chloride) is currently unknown and worth further study by, for example, employing compounds isolated from cyanobacteria, spent-medium from cyanobacterial cultures, or cyanobacterial extracts/lysates.

Hypotheses regarding the tolerance of cyanobacteria to perchlorate stress along with future research that will be required to test them are summarized in Table 3. Although the present study provides an insight into the possible tolerance of cyanobacteria to perchlorates at the concentration found on the martian surface, some study limitations must be stressed. The perchlorate exposure was conducted under otherwise optimal conditions for cyanobacterial growth, whereas on Mars, the perchlorate would constitute one of the challenging factors for their survival.

Moreover, UVC radiation has been shown to further activate perchlorate to act as a powerful oxidant and increase its toxic effects on microorganisms (Wadsworth and Cockell, 2017). In turn, gamma-radiolyzed perchlorate generates increased levels of hydroxyl radicals and hydrogen peroxide, which can exacerbate its damaging effect (Georgiou *et al.*, 2017). On the other hand, if perchlorate can be transformed to oxidation agents under martian conditions as experimentally shown (Crandall *et al.*, 2017), then the survival of cyanobacteria that are adapted to effectively mitigate the oxidative stress would be favored.

Finally, the present study employed only magnesium perchlorate, whereas the martian surface can also contain sodium and calcium perchlorates in differing proportions (Hecht *et al.*, 2009; Glavin *et al.*, 2013; Hassler *et al.*, 2014; Kounaves *et al.*, 2014; Ojha *et al.*, 2015). Altogether, the presented findings should be treated as preliminary and as a foundation for more in-depth, detailed studies on the survival of cyanobacteria under martian conditions.

## 5. Conclusions

Results of this experimental study indicate that perchlorates adversely affect the growth of cyanobacteria, although some species, that is, *C. thermalis*, *L. foveolarum*, *A. africanum*, *G.* cf. *acuminatum*, and *C. komarekiana*, can maintain growth despite concentrations of magnesium perchlorate being as high as 1% (6.0 m*M* of ClO_4_^−^). The reduction in growth rates is likely a result of the perchlorate-induced generation of reactive oxygen species, which leads to oxidative stress, as evidenced by increased peroxidation of lipids. Shifts in carotenoid pigments may offer, at least in part, protection against perchlorates in cyanobacteria.

It is plausible that the tolerance observed in the present study is the indirect effect of cyanobacterial adaptation to extreme conditions such as high salinity-, desiccation-, or UV-induced oxidation. Further studies should focus on the biochemical and molecular basis of perchlorate tolerance in cyanobacteria. Their elucidation is important for potential genetic engineering and adaptation of other organisms to the martian environment and for use as components of Mars life support systems.

## Abbreviations Used

MDA: malondialdehyde
SD: standard deviation
SOD: superoxide dismutase
TBA: thiobarbituric acid
UV: ultraviolet
